# Childhood Mental Health Characteristics of Adults Diagnosed With Borderline Personality Disorder: A Descriptive Study

**DOI:** 10.7759/cureus.101024

**Published:** 2026-01-07

**Authors:** Diana M Wang, Catherine A Ha, Joshua Z Xian, Henry H Kim, Gino A Mortillaro

**Affiliations:** 1 Department of Psychiatry, Kaiser Permanente Fontana Medical Center, Fontana, USA; 2 School of Medicine, University of California, San Francisco, USA; 3 Department of Psychiatry and Behavioral Sciences, Boston Children's Hospital, Boston, USA

**Keywords:** borderline personality disorder (bpd), child and adolescent psychiatry, healthcare resource utilisation, psychiatric co-morbidities, psychiatry and mental health

## Abstract

Introduction: Borderline personality disorder (BPD) is a burdensome and diagnostically challenging condition. There is a need to identify its risk factors, yet prior studies are scarce and small-scale. We aimed to describe the prevalence of psychiatric comorbidities and the level of mental health resource utilization during adolescence in patients with BPD.

Methods: We conducted a retrospective descriptive study of 938 participants (aged 18-26 years) with BPD at a large, multi-site, integrated health maintenance organization (HMO) system in Southern California. Data were collected from January 2010 to September 2020. Comorbidities of interest were other psychiatric conditions diagnosed before age 18. Healthcare utilization data included age of first psychiatry encounter, age at first psychiatric hospitalization, number of psychiatric hospitalizations per year, number of sessions of intensive outpatient group therapy and partial hospitalization, number of visits with a psychiatrist, number of crisis calls, and number of psychotherapy sessions.

Results: The analytic cohort included 816 (87.0%) female patients with a mean age of 22 years. BPD prevalence in the overall population was 0.1% (n=2,287). Comorbidities with the highest prevalence included depressive and anxiety disorders at 83.5% (n=783) and 69.6% (n=653), respectively. Suicidal ideation and suicide attempts were prevalent at 38.6% (n=362) and 0.5% (n=5). Before the age of 18 years, 70.7% (n=663) had a psychiatric visit, 86.7% (n=813) had psychotherapy, and 34.8% (n=326) had a psychiatric hospitalization.

Conclusion: The findings of this study suggest high psychiatric comorbidity and healthcare utilization in patients with BPD. Given lower rates of adolescent suicidality, early intervention may decrease BPD-associated mortality. Psychiatric comorbidities may predict the risk of developing BPD. Future comparative analyses should validate the statistical significance of these predictors and whether screening improves BPD outcomes.

## Introduction

Borderline personality disorder (BPD) is a serious psychiatric condition defined as a “pervasive pattern of instability of interpersonal relationships, self-image, and affects, and marked impulsivity, beginning by early adulthood and present in a variety of contexts” [1]. However, due to the wide range and severity of BPD symptoms, vague descriptors, and variability in disease course, debate continues over the appropriate age for diagnosis, the criteria for diagnosis, and the necessity of BPD as a diagnosis altogether [2,3]. Compared to other psychiatric illnesses, BPD has been associated with higher rates of suicide, severe psychological impairment, and utilization of mental health resources [4,5]. While BPD affects up to 3% of the general population, patients with BPD encompass 10% of outpatient mental health visits and 20% of psychiatric inpatient hospitalizations [1,6]. The high mortality, morbidity, and burden of BPD call for the need to identify risk factors for its development, but few studies have examined traits in childhood that may predict BPD diagnosis.

While diagnosing BPD before age 18 has been historically controversial, there has been evidence of similar reliability and stability of BPD in adolescence and adulthood, thus supporting earlier diagnosis [7,8]. Previous research has identified impulsivity, emotional sensitivity, over-reactivity, invalidating parenting, disorganized attachment, and childhood trauma as potential pediatric predictors of BPD [9]. Prior studies have also suggested that adolescents with BPD have comorbid mental health conditions, most notably mood disorders, eating disorders, dissociative and post-traumatic stress disorders, substance use disorders, and oppositional defiant disorder [10,11]. However, these studies have been on relatively smaller scales, and the number of factors investigated has been limited. Our study aims to describe the prevalence of psychiatric comorbidities and the level of mental health resource utilization in adolescent patients with BPD within a large health maintenance organization (HMO) health system.

## Materials and methods

This was a retrospective descriptive chart review study at a large, multi-site, integrated HMO health system, Kaiser Permanente Fontana Medical Center, Fontana, in Southern California, United States that serves a population of both privately and publicly insured patients from data collected between January 2010 and September 2020. The study was approved by the Institutional Review Board of Kaiser Permanente Southern California/Hawaii (approval number: 12709).

Study population

**Figure 1:**
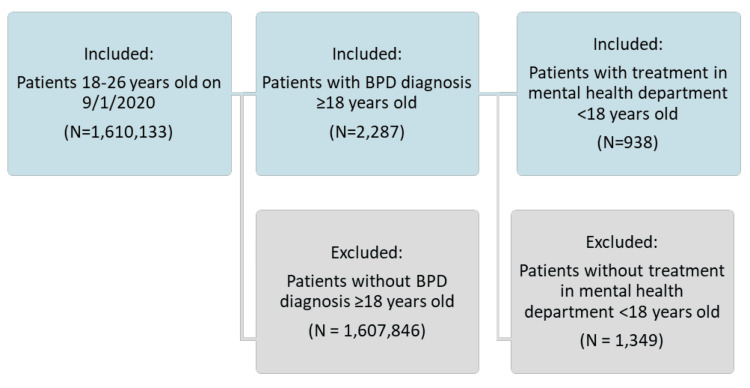
Study cohort selection flowchart.

Out of 1,610,133 patients aged 18 to 26 years, those without a BPD diagnosis and those without treatment in the mental health department prior to age 18 were excluded, resulting in a final study population of 938 patients (Figure 1).

Data collection

Age, race, sex, psychiatric diagnoses, and healthcare utilization data were retrospectively retrieved from patient medical records. Psychiatric illnesses were identified via International Classification of Diseases (ICD)-9 and ICD-10 codes and grouped into broader categories based on the Diagnostic and Statistical Manual of Mental Disorders, Fifth Edition, Text Revision (Table 1) [1].

**Table 1:** Diagnostic criteria for borderline personality disorder from the DSM-5-TR DSM-5-TR: Diagnostic and Statistical Manual of Mental Disorders, Fifth Edition, Text Revision [1].

DSM-5-TR Criteria
A pervasive pattern of instability of interpersonal relationships, self-image, and affects, and marked impulsivity, beginning by early adulthood and present in a variety of contexts, as indicated by five (or more) of the following:
1. Frantic efforts to avoid real or imagined abandonment. (Note: Do not include suicidal or self-mutilating behavior covered in Criterion 5).
2. A pattern of unstable and intense interpersonal relationships characterized by alternating between extremes of idealization and devaluation.
3. Identity disturbance: markedly and persistently unstable self-image or sense of self.
4. Impulsivity in at least two areas that are potentially self-damaging (e.g., spending, sex, substance abuse, reckless driving, binge eating). (Note: Do not include suicidal or self-mutilating behavior covered in Criterion 5).
5. Recurrent suicidal behavior, gestures, or threats, or self-mutilating behavior.
6. Affective instability due to a marked reactivity of mood (e.g., intense episodic dysphoria, irritability, or anxiety usually lasting a few hours and only rarely more than a few days).
7. Chronic feelings of emptiness.
8. Inappropriate, intense anger or difficulty controlling anger (e.g., frequent displays of temper, constant anger, recurrent physical fights).
9. Transient, stress-related paranoid ideation or severe dissociative symptoms.

Healthcare utilization data included age at first encounter with psychiatry, age at first psychiatric hospitalization, number of psychiatric hospitalizations per year, number of sessions of intensive outpatient group therapy and partial hospitalization, number of visits with a psychiatrist, number of crisis calls, and number of psychotherapy sessions. Data were reported as the number of relevant encounters per year to control for the fact that these patients may not have had services through our specific healthcare organization for their entire life, and the electronic health record was only fully established throughout this hospital system in 2010.

## Results

Demographics

Of the 938 participants included in the study population, 816 (87.0%) were female, with a mean age of 22 years (Table 2). A total of 395 (42.1%) participants were of Hispanic ethnicity, 378 (40.3%) were of White race, 83 (8.8%) were of Black race, 47 (5.0%) were Asian, three (0.3%) were Pacific Islander, two (0.2%) were Native American or Alaskan, 15 (1.6%) were multiracial, 10 (1.1%) were other, and five (0.5%) were of unknown race. Compared to the overall patient population, the study population had a higher proportion of female and White participants, a lower proportion of Asian participants, and a lower mean age. The prevalence of BPD in the overall population was 0.1% (2,287 out of 1,610,133).

**Table 2:** Study population demographics by age, age group, sex, and race/ethnicity (N=938)

Demographic Characteristics	Values
Age (years)	
Mean	21.9
Median	22
Range	18.0-26.0
Age Group (years), n (%)	
18	66 (7%)
19	105 (11.2%)
20	116 (12.4%)
21	120 (12.8%)
22	139 (14.8%)
23	124 (13.2%)
24	110 (11.7%)
25	93 (9.9%)
26	65 (6.9%)
Sex, n (%)	
Female	816 (87%)
Male	122 (13%)
Race/Ethnicity, n (%)	
White	378 (40.3%)
Asian	47 (5%)
Black	83 (8.8%)
Hispanic	395 (42.1%)
Multiple	15 (1.6%)
Native American or Alaskan	2 (0.2%)
Pacific Islander	3 (0.3%)
Other	10 (1.1%)
Unknown	5 (0.5%)

Comorbidities

In examining comorbid conditions diagnosed before age 18, 219 (23.3%) were diagnosed with attention-deficit/hyperactivity disorder (ADHD), 653 (69.6%) with anxiety disorders, 179 (19.1%) with bipolar disorders, 123 (13.1%) with disruptive behavior disorders, 783 (83.5%) with depressive disorders, 70 (7.5%) with neurodevelopmental disorders, 39 (4.2%) with dissociative, conversion, and factitious disorders, 132 (14.1%) with feeding and eating disorders, 29 (3.1%) with gender dysphoria, 60 (6.4%) with impulse-control disorders, zero (0%) with paraphilic disorders, 53 (5.7%) with personality disorders, 58 (6.2%) with schizophrenia spectrum and other psychotic disorders, five (0.5%) with suicide attempt, 184 (19.6%) with self-harm behavior, 362 (38.6%) with suicidal ideation, 52 (5.5%) with sleep-wake disorders, 343 (36.6%) with trauma- and stress-related disorders, 222 (23.7%) with substance-related and addictive disorders, 13 (1.4%) with tic disorders, 207 (22.1%) with other or unspecified mood disorders, and 93 (9.9%) with unspecified nonpsychotic disorders (Table 3).

**Table 3:** Concurrent diagnoses during adolescence (age < 18 years old) in patients with BPD (N=938) BPD: borderline personality disorder; ADHD: attention deficit hyperactivity disorder

Diagnoses	Frequency (Percentage)
ADHD	
No	719 (76.7%)
Yes	219 (23.3%)
Anxiety Disorders	
No	285 (30.4%)
Yes	653 (69.6%)
Bipolar and Related Disorders	
No	759 (80.9%)
Yes	179 (19.1%)
Disruptive Behavior Disorders	
No	815 (86.9%)
Yes	123 (13.1%)
Depressive Disorders	
No	155 (16.5%)
Yes	783 (83.5%)
Neurodevelopmental Disorders	
No	868 (92.5%)
Yes	70 (7.5%)
Dissociative, Conversion, and Factitious Disorders	
No	899 (95.8%)
Yes	39 (4.2%)
Feeding and Eating Disorders	
No	806 (85.9%)
Yes	132 (14.1%)
Gender Dysphoria	
No	909 (96.9%)
Yes	29 (3.1%)
Impulse-Control Disorders	
No	878 (93.6%)
Yes	60 (6.4%)
Paraphilic Disorders	
No	938 (100%)
Yes	0 (0%)
Personality Disorders	
No	885 (94.3%)
Yes	53 (5.7%)
Schizophrenia Spectrum and Other Psychotic Disorders	
No	880 (93.8%)
Yes	58 (6.2%)
Suicide Attempts (initial encounter)	
No	933 (99.5%)
Yes	5 (0.5%)
Self-Harm and Suicide Attempts	
No	754 (80.4%)
Yes	184 (19.6%)
Suicidal Ideation	
No	576 (61.4%)
Yes	362 (38.6%)
Sleep-Wake Disorders	
No	886 (94.5%)
Yes	52 (5.5%)
Trauma- and Stress-Related Disorders	
No	595 (63.4%)
Yes	343 (36.6%)
Substance-Related and Addictive Disorders	
No	716 (76.3%)
Yes	222 (23.7%)
Tic Disorders	
No	925 (98.6%)
Yes	13 (1.4%)
Other and Unspecified Mood Disorders	
No	728 (77.9%)
Yes	207 (22.1%)
Unspecified Nonpsychotic Disorders	
No	845 (90.1%)
Yes	93 (9.9%)

Healthcare utilization

Of the 938 participants, 935 (99.7%) had at least one psychiatric encounter in adolescence or adulthood, with a mean age at the first encounter with psychiatry of 15 years (Table 4). A total of 584 (62.3%) had at least one psychiatric hospitalization in adolescence or adulthood, with a mean age at first inpatient psychiatric hospitalization of 17 years. The mean time between participants’ first psychiatric encounter and first inpatient psychiatric hospitalization was two years. Before age 18, 663 (70.7%) participants had a psychiatric visit with a mean of 4.36 psychiatry visits per year, 813 (86.7%) had psychotherapy visits with a mean of 5.24 per year, and 326 (34.8%) had a psychiatric hospitalization with a mean of 2.07 per year. Prior to adulthood, 315 (33.6%) were part of an intensive outpatient therapy program (IOP) or partial hospitalization program (PHP) visit with a mean of 8.51 per year, and 61 (6.5%) had psychiatric crisis encounters with a mean of 1.84 per year.

**Table 4:** Mental health service utilization data in study population (based only on those who utilized each service) (N=938) *Number of patients who utilized this healthcare service **Includes patients who utilized this healthcare service in adolescence and/or adulthood IOP: intensive outpatient program; PHP: partial hospitalization program

	Frequency* (Percentage)	Minimum	Median	Maximum	Mean	Standard Deviation
Age at First Psychiatric Encounter (years)	935** (99.7%)	7.00	15.00	22.00	15.00	2.09
Age at First Psychiatric Hospitalization (years)	584** (62.3%)	10.00	17.00	25.00	17.20	2.70
Psychiatric Prescriber Visits Per Year Before Age 18	663 (70.7%)	0.20	3.39	52.18	4.36	4.64
Number of IOP/PHP Encounters Per Year Before Age 18	315 (33.6%)	0.17	3.91	162.33	8.51	13.56
Number of Crisis Encounters Per Year Before Age 18	61 (6.5%)	0.24	0.84	17.39	1.84	2.77
Number of Psychotherapy Visits Per Year Before Age 18	813 (86.7%)	0.12	2.61	182.63	5.24	10.23
Number of Psychiatric Hospitalizations Per Year Before Age 18	326 (34.8%)	0.13	1.17	73.05	2.07	4.82

## Discussion

Our descriptive study looked at early adolescent comorbidities and psychiatric service utilization among patients with BPD in a large HMO health system. 

Comorbidities

The comorbidities with the highest prevalences in our study population before age 18 were depressive and anxiety disorders at 83.5% (n=783) and 69.6% (n=653), respectively, which is consistent with prior literature on both adolescents and adults [10,12]. Other previously indicated comorbid adolescent diagnoses, including trauma- and stress-related disorders, substance-related and addictive disorders, attention-deficit/hyperactivity disorder (ADHD), and feeding and eating disorders, were also notable in our study, with prevalences of 36.6% (n=343), 23.7% (n=222), 23.3% (n=219), and 14.1% (n=132), respectively [10,13]. Our data reaffirm these comorbidities as potential early predictors of BPD to be explored in future comparative analyses.

Additionally, about 60% of patients with BPD have been shown to exhibit suicidality or self-injurious behavior, and BPD symptom severity has been associated with suicidal ideation and attempts in adolescents [1,14]. One prior study found BPD to be associated with increased suicidal ideation and self-injury in children as early as preschool [9]. Our data show suicidal ideation and self-harm in 38.6% (n=362) and 19.6% (n=184) of the study population before age 18, respectively, both of which are at relatively high prevalences among psychiatric comorbidities. Interestingly, however, only 0.5% (n=5) of participants had attempted suicide in adolescence, a much lower number than other measures of suicidality. The literature is limited in specific analyses of adolescent suicide attempts in patients with BPD, but our data suggest that early intervention may help prevent progression of suicidal ideation and self-harm to active suicide attempt. Age-adapted psychotherapy approaches, such as dialectical behavioral therapy and mentalization-based therapy, have been shown to improve borderline symptoms, suicidal behaviors, and self-injury associated with BPD in adolescence [15,16]. Of note, the overall suicide rate in our study population in adolescence or adulthood was 3.3% (n=31), which is much lower than that reported in other clinical populations at 8-10% [17]. This difference may reflect differences in documentation, cohort characteristics, or patterns of healthcare access in our cohort; however, other measures of suicidality, such as suicidal ideation and self-injury, are prevalent. Thus, early intervention may still be key to potentially decreasing BPD-associated mortality.

Healthcare utilization

Patients with BPD have demonstrated high health care and emergency room utilization, especially in the setting of crises, self-injury, and suicidality [18,19]. The psychiatric healthcare utilization in our study population reflected this; the majority of patients with BPD had received psychiatric services before age 18. Stratifying for specific visit types, 70.7% (n=663) of patients had psychiatric visits, 86.7% (n=813) had psychotherapy visits, 34.8% (n=326) had psychiatric hospitalizations, and 33.6% (n=315) had an IOP or PHP encounter before adulthood. These data suggest that high healthcare utilization is seen even at earlier ages in patients with BPD.

Limitations and future directions

While these findings support the need for further research in pediatric personality disorders, there are a few limitations. Our study was descriptive and did not use statistical methods to analyze associations between BPD and potential predictors. Instead, we reported prevalences of comorbidities and healthcare utilization that are notable within our study population and should be further investigated in future research.

Additionally, due to changes between ICD-9 and ICD-10, certain diagnostic codes from the prior version needed to be equated to those in the current one. For example, non-suicidal self-harm behaviors and suicide attempts were combined under the same ICD-9 code but later separated in ICD-10 coding. Thus, our study may not fully capture specific distinctions between different code categorizations.

Furthermore, the generalizability of our findings remains limited to HMO-health-system clinical settings in the United States. The prevalence of BPD diagnoses in our study population was 0.1%, which is relatively low compared to the 3% previously noted in the general population [6]. This may also reflect underdiagnosis, given the challenges of diagnosing BPD that include wide variability of disease symptoms and course, potential overlap of traits with other psychiatric illnesses, and hesitancy to diagnose a condition tied to a stigma of therapeutic difficulty [2,20].

Future studies should perform comparative analyses focused on pediatric populations to see if notable adolescent comorbidities identified in our study, such as depression and anxiety, are associated with a greater risk of developing BPD, and if early screening for these factors can improve BPD-associated outcomes. Further research should also specifically consider socio-cultural factors to elucidate whether potential disparities between different demographic groups in adolescence are reflective of those observed in adulthood. For instance, African American women with BPD were shown to experience more severe symptoms of distress tolerance than White counterparts, and BPD was found to be more prevalent among Native American men, younger adults, and those with lower socioeconomic status [21,22]. Understanding these patterns in adolescents is essential to provide equitable care. In addition, more studies on potential early interventions in adolescents are warranted to identify effective ways to reduce the incidence of BPD, BPD-associated suicidality, and the severity of borderline traits.

## Conclusions

BPD continues to pose multifaceted challenges in the identification, treatment, and management of symptoms in both the adult and child and adolescent patient populations. Early diagnosis and treatment of BPD may be essential to improving outcomes later in life. This study further elucidated the high comorbidity of mood disorders and high healthcare utilization by this patient population, though further research is needed to validate the statistical significance of these potential predictors.
